# Exploring the Potential of *Nannochloropsis* sp. Extract for Cosmeceutical Applications

**DOI:** 10.3390/md19120690

**Published:** 2021-12-02

**Authors:** Sun Young Kim, Yong Min Kwon, Kyung Woo Kim, Jaoon Young Hwan Kim

**Affiliations:** National Marine Biodiversity Institute of Korea, Jangsan-ro 101-75, Seocheon-gun 33662, Chungcheongnam-do, Korea; dydn5588@mabik.re.kr (S.Y.K.); jichi9@mabik.re.kr (Y.M.K.); kimkw79@mabik.re.kr (K.W.K.)

**Keywords:** *Nannochloropsis*, antioxidant, anti-melanogenic, skin-moisturizing, UV protection, anti-aging

## Abstract

Recently, there has been emerging interest in various natural products with skin protective effects as they are recognized as safe and efficient. Microalgae have developed chemical defense systems to protect themselves against oxidative stress caused by UV radiation by producing various bioactive compounds including a number of secondary metabolites, which have potential for cosmeceutical applications. In addition, microalgae have various advantages as a sustainable source for bioactive compounds with diverse functions due to their rapid growth rate, high productivity, and use of non-arable land. In this study, we aimed to investigate the cosmeceutical potential of ethanol extract from *Nannochloropsis* sp. G1-5 (NG15) isolated from the southern West Sea of the Republic of Korea. It contained PUFAs (including EPA), carotenoids (astaxanthin, canthaxanthin, β-carotene, zeaxanthin, violaxanthin), and phenolic compounds, which are known to have various skin protective functions. We confirmed that the NG15 extract showed various skin protective functions with low cytotoxicity, specifically anti-melanogenic, antioxidant, skin-moisturizing, anti-inflammatory, anti-wrinkling, and UV protective function, by measuring tyrosinase inhibition activity; melanin content; DPPH radical scavenging activity; expression of *HAS-2*, *MMP-1*, and *Col1A1* genes; and elastase inhibition activity as well as cell viability after UV exposure. Our results indicated that the NG15 extract has the potential to be used for the development of natural cosmetics with a broad range of skin protective functions.

## 1. Introduction

The skin is the largest organ, covering the entire human body and providing a physical barrier to protect the internal tissues from external stimuli such as ultraviolet (UV) radiation, chemicals, pathogens, and physical stresses [1,2]. In addition, it has physiologically important roles in helping to sustain organisms, including the preservation of water, immunological functions, sensory perception, and the regulation of body temperature [3,4]. The skin consists of three layers: the epidermis, dermis, and hypodermis. In the epidermis, keratinocytes, Langerhans, melanocytes, and sebaceous glands play essential roles for the repair of skin damage, determining the skin color, protecting skin from UV radiation, providing immunity, and lubricating the skin. In the dermis, located beneath the epidermis, predominant fibroblast cells produce collagen and elastic fibers. Collagen provides tensile strength and toughness to resist deformation, whereas elastin provides elasticity and flexibility, allowing tissues to return to their original shape upon removal of the deforming force. Collagen and elastin are cross-linked to provide structural support for the skin [4,5,6,7]. Hyaluronic acid (HA), a glycosaminoglycan, is a major component of the extracellular matrix (ECM). HA is a key molecule for the retention of skin moisture due to its capacity to bind and retain water molecules up to 1000 times its weight [8,9]. HA forms a gel by absorbing water and provides tissues with resistance to compression. The hypodermis, which is composed of primarily fat and loose connective tissue, stores energy and provides insulation to the body [5,6].

Skin aging is a complex biological process induced by intrinsic and extrinsic factors [10,11]. Intrinsic aging is an inevitable process caused by physiological and genetic changes with the passage of time, resulting in fine wrinkles, gradual dermal atrophy, and thin and dry skin [10,12,13]. Extrinsic aging is engendered by cumulative exposure to environmental factors including UV radiation, smoking, and air pollution. These environmental factors are known to boost physiological and morphological alterations of skin, resulting in premature skin aging [12,13,14]. Among environmental factors, exposure to UV radiation is the primary reason for extrinsic skin aging and is referred to as photoaging [14,15,16]. Different from intrinsically aged skin, prematurely photoaged skin usually shows coarseness, irregular, mottled pigmentation, a thickened epidermis, deep wrinkles, laxity, and roughness [5,10,17]. Even though intrinsic skin aging and extrinsic skin aging are induced by different factors, both share similar molecular mechanisms. In particular, reactive oxygen species (ROS) are generated by oxidative cell metabolism and play a key role in both processes [18]. ROS trigger the activation of mitogen-activated protein kinases (MAPK) and subsequent nuclear factor-κB (NF-κB) and transcription factor activator protein-1 (AP-1), which lead to upregulation of metalloproteinases (MMPs: MMP-1, MMP-3, and MMP-9) and downregulation of procollagen-1, resulting in the reduction of collagen content in aged skin [2,19]. ROS can also activate blood neutrophils to infiltrate the skin and secrete elastase, which degrades elastic fibers to lose skin elasticity [20]. Skin aging is also strongly related to the loss of skin moisture. It has been reported that a marked reduction of HA in the epidermis as a result of skin aging results in the loss of skin moisture [8,21]. HA is synthesized by hyaluronan synthases (HAS; HAS-1, -2, -3,) in epidermal keratinocytes and dermal fibroblasts [8]. Melanin is produced by conversion of L-tyrosine to L-dihydroxyphenylalanine (L-DOPA), which is catalyzed by tyrosinase. Melanin is responsible for skin color and plays an important role in protecting skin from damage by exposure to sunlight. However, the accumulation of ROS increases the activity of tyrosinase, leading to hyperpigmentation such as age spots and melasma due to excessive production of melanin. Therefore, hyperpigmentation can be alleviated by free radical scavenger and tyrosinase inhibitors [22,23].

In the last decades, strategies for healthy aging have been mandatory due to increased life expectancy. In addition, there is growing interest in maintaining a young and beautiful appearance because skin health and beauty are perceived as important biological factors representing human well-being. It is also regarded that a youthful and beautiful appearance may have a positive effect on social behavior [10,13]. Therefore, millions of consumers use cosmetic skin care products daily, and the global market of cosmetic products is projected to reach $805 billion by 2023, at an estimated growth rate of 7.14% per year from 2018 to 2023 [24,25]. Due to the adverse side effects of synthetic cosmetic products, there is a growing demand to use natural products, including molecules from plants, animals, and marine organisms. Cosmeceuticals are derived from cosmetics and pharmaceuticals, which refer to cosmetic products containing bioactive ingredients with functions of UV protection, skin whitening, anti-wrinkling, and anti-aging [26]. In the cosmetic industry, cosmeceuticals are the fastest growing sector and natural products are emerging as a novel source of potential bioactive substances for cosmeceutical applications [25].

Microalgae are prokaryotic or eukaryotic photosynthetic microorganisms that can grow rapidly and survive in extreme environmental conditions (e.g., temperature variation, anaerobiosis, salinity, photooxidation, osmotic pressure, and UV radiation) [27]. Microalgae are also superior to terrestrial plants due to high productivity, limited seasonal variation, easier extraction, and abundant raw materials [27]. Microalgae are constantly exposed to environmental stresses. Thus, they evolved to develop various strategies such as ultrastructural, physiological, and biochemical changes [28]. The natural products derived from microalgae with potential for cosmetic or cosmeceutical purposes include photosynthetic pigments, lipids, phenolic compounds, amino acids, peptides, carbohydrates, and vitamins [25]. Among natural products, carotenoids, which are known as strong antioxidants and free radical scavengers, can be utilized for anti-aging and photoprotection purposes [29]. Among carotenoids, astaxanthin, which exhibits a higher antioxidant activity than β-carotene and ascorbic acid, has an interesting depigmentation function that can protect skin from age spots by reducing melanin synthesis by 40% [30,31]. Zeaxanthin and other carotenoids also show the activities of UV absorption and tyrosinase inhibition [32]. Microalgae often contain high lipid contents including polyunsaturated fatty acids (PUFAs). PUFAs play an important role in photoprotection, maintaining membrane fluidity and preventing intracellular ice crystal formation, which enables survival in extreme environmental conditions such as high light intensity, UV radiation, and low temperature [33,34]. Omega-3 fatty acids such as docosahexaenoic acid (DHA) and eicosapentaenoic acid (EPA) have positive effects in attenuating skin photoaging by suppression of UV-induced metalloproteinases (MMPs) and anti-inflammatory activities [35]. It has been also reported that γ-linolenic acid has some cosmetic effects such as revitalizing the skin and slowing the aging process, and linoleic acid is used for the treatment of hyperplasia of the skin [36]. Phenolic compounds from microalgae are known to exhibit diverse activities such as antioxidant, antiallergic, anti-inflammatory, and UV protection functions [2,37]. 

*Nannochloropsis* species, the small unicellular eustigmatophycean algae, are recognized for their high lipid content and high photoautotrophic biomass productivity, as well as for their successful cultivation at a large scale [38]. *Nannochloropsis* has been exploited as a source of unsaturated fatty acids (including EPA) for the diet of aquaculture-raised marine invertebrates. They also contain phenolic compounds, vitamins, and various pigments with antioxidant activities [39]. In addition, they do not produce toxins and their toxicological safety has been proven by their long-term use as food for marine fish and shellfish larvae [40]. Although they have diverse valuable bioactive compounds, there are few reports on the application of *Nannochloropsis* for cosmetic and cosmeceutical purposes [41]. Considering that biochemical composition of microalgae affecting cosmetic effects can be different even among isolates of the same species [42], further investigation of this microalgal species is needed to satisfy the increasing demand for natural and safe cosmetic products. In this study, we investigated the cosmeceutical potential of extract from *Nannochloropsis* sp. G1-5 (NG15) isolated from the southern West Sea of the Republic of Korea. We analyzed its various biological activities, including its anti-melanogenic, antioxidant, skin-moisturizing, anti-inflammatory, anti-wrinkling, and UV protection functions. We further analyzed its biochemical content and the composition of fatty acids, carotenoids, and phenolic compounds.

## 2. Results

### 2.1. Isolation of Nannochloropsis sp. G1-5 and Analysis of Biochemical Composition

In this study, we isolated a colony with a brownish color indicating the presence of pigments of carotenoids and polyphenols, which was obtained by spreading sea water samples onto F/2 agar plates. We identified the isolate using 18S rDNA PCR followed by sequencing and named it as *Nannochloropsis* sp. G1-5 (NG15). After cultivation of NG15 and extraction using ethanol, the composition and content of fatty acid methyl esters (FAMEs), carotenoids, and phenolic compounds in the NG15 extract were determined. The total contents of fatty acids, carotenoids, phenolics, and flavonoids in the crude extract were 58.2%, 1.6%, 7.7%, and 2.0%, respectively (Table 1, Table 2 and Table 3). Specifically, the crude extract contained various PUFAs in the lipid content like γ-linolenic, linoleic acid, and EPA, which have anti-aging, photoprotection, anti-inflammatory activities (Table 1). It also had various carotenoid compounds including astaxanthin, β-carotene, zeaxanthin, canthaxanthin, and violaxanthin (Table 2, Figure S1, Table S1), which were known to have antioxidant, photoprotective, and anti-melanogenic functions. In addition, total phenolics’ and flavonoids’ contents in the crude extract of NG15 (Table 3) were relatively high compared to those of other previously reported microalgae [43,44,45]. 

### 2.2. In Vitro Cytotoxicity of NG15 Extract

**Figure 1:**
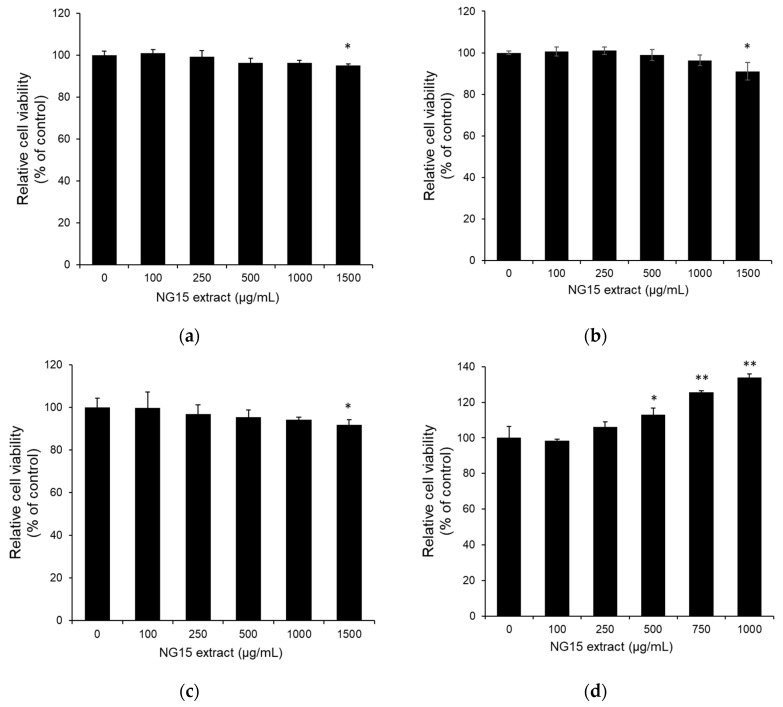
The effect of NG15 extract (0–1500 μg/mL) on the viability of (**a**) B16F10, (**b**) NHDF, (**c**) NF-κB luciferase reporter NIH3T3 stable cells, and (**d**) CCD-986sk cells. Control means the cell viability of cells without the NG15 extract; * denotes a *p* value < 0.05, *n* = 3, Student’s *t* test; ** denotes a *p* value < 0.01, *n* = 3, Student’s *t* test.

The effect of the NG15 extract on the viability of B16F10, CCD-986sk, normal human dermal fibroblast (NHDF), and NF-κB luciferase reporter NIH3T3 stable cells was determined by MTT assay at a concentration of 100–1500 μg/mL. The treatment with the NG15 extract did not significantly reduce the cell viability of all cell lines tested up to the concentration of 1000 μg/mL, but it slightly decreased at 1500 μg/mL (Figure 1a–d).

### 2.3. Anti-Melanogenic Activity of NG15 Extract

**Figure 2:**
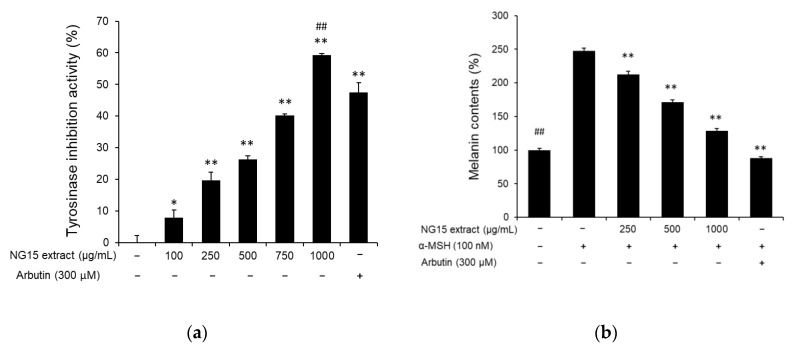
The anti-melanogenic effect of NG15 extract. (**a**) In vitro tyrosinase inhibition activity of NG15 extract against mushroom tyrosinase. ** denotes a *p* value < 0.01 versus a normal (untreated) group. ## denotes a *p* value < 0.01 versus arbutin-treated cells; *n* = 3; Student’s *t* test. (**b**) The effect of NG15 extract on melanin contents in α-melanocyte-stimulating hormone (MSH)-stimulated B16F10 cells. ## and ** denote a *p* value < 0.01 versus α-MSH-only-treated cells; *n* = 3; Student’s *t* test. Arbutin (300 μM) was used as a positive control.

To evaluate whether the NG15 extract has a skin-whitening effect, we analyzed its influence on tyrosinase activity and intracellular melanin synthesis. From the tyrosinase inhibition assay, we observed that tyrosinase activity was reduced by treatment with the NG15 extract in a dose-dependent manner. The tyrosinase inhibition activities were 7.84 ± 2.54, 19.68 ± 2.64, 26.24 ± 1.21, 40.16 ± 0.55, and 59.2 ± 0.48% after treatment with the NG15 extract at a concentration of 100, 250, 500, 750, and 1000 μg/mL, respectively, while arbutin (300 μM), used as a positive control, exhibited tyrosinase inhibition activity of 47.52 ± 3.09% (Figure 2a). The tyrosinase inhibition activity of the NG15 extract at 1000 μg/mL was higher than that of arbutin (300 μM). To determine the anti-melanogenic activity of the NG15 extract, The B16F10 cells were stimulated with α-melanocyte-stimulating hormone (α-MSH, 100 nM) and melanin contents in the B16F10 cells treated with the NG15 extract or arbutin were measured. The NG15 extract exhibited a significant inhibitory effect on the melanin synthesis in a dose-dependent manner. Compared to non-treated cells, the melanin contents after treatment of the extract were 212.53 ± 4.72, 171.06 ± 3.57, and 128.60 ± 3.67% at a concentration of 250, 500, and 1000 μg/mL, respectively. The melanin content of B16F10 cells treated with only α-MSH (100 nM) showed 247.65 ± 4.15%. This result represents the fact that the NG15 extract decreased melanin content in α-MSH-treated B16F10 cells up to 48%. The positive control, arbutin (300 μM), showed 88.27 ± 1.50% of melanin content compared to non-treated cells, corresponding to a 64% reduction of melanin content in α-MSH-treated B16F10 cells (Figure 2b).

### 2.4. Antioxidant, Anti-Inflammatory, and UV-Protection Activities of NG15 Extract

**Figure 3:**
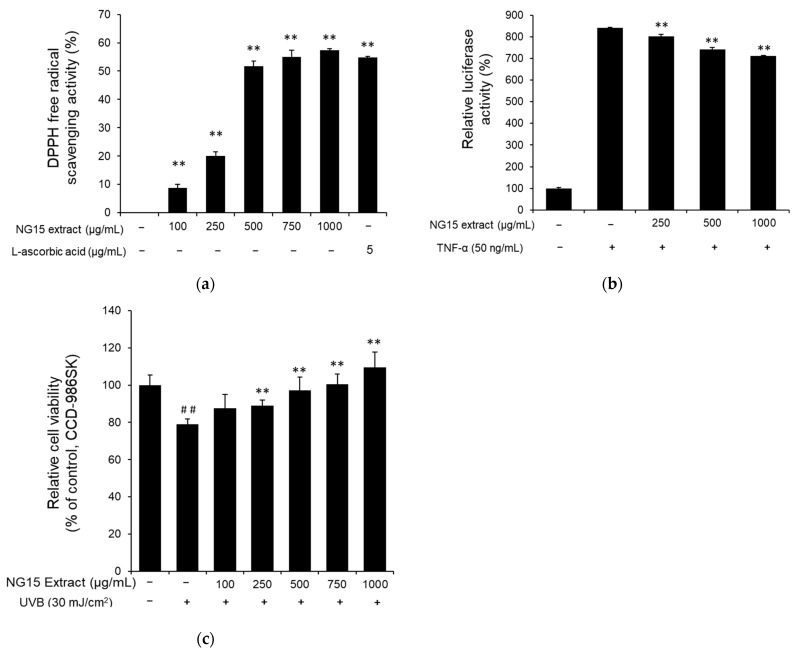
Antioxidant, anti-inflammatory, and UV-protection activities of NG15 extract. (**a**) DPPH free radical scavenging activity of NG15 extract. L-ascorbic acid (5 μg/mL) was used as a positive control. ** denotes a *p* value < 0.01 versus a normal (untreated) group. *n* = 3; Student’s *t* test. (**b**) The effect of NG15 extract on NF-κB-dependent, TNF-α-induced luciferase activity in NF-κB luciferase reporter NIH3T3 stable cells. ** denotes a *p* value < 0.01 versus TNF-α-only-treated cells. *n* = 3; Student’s *t* test. (**c**) The protective effect of NG15 extract on the viability of CCD-986sk cells after exposure to UVB (30 mJ/cm^2^). ## denotes a *p* value < 0.01 versus a normal (untreated and unexposed to UVB) group. ** denotes a *p* value < 0.01 versus UVB-only-treated group. *n* = 3; Student’s *t* test.

The DPPH assay was employed to evaluate antioxidant activity of the NG15 extract. The DPPH radical scavenging activity of the NG15 extract increased in a dose-dependent manner at a concentration range of 100–1000 μg/mL. Specifically, DPPH radical scavenging activities were 8.71 ± 1.38, 20.12 ± 1.38, 51.65 ± 1.88, 54.95 ± 2.38, and 57.36 ± 0.52% after treatment with 100, 250, 500, 750, and 1000 μg/mL of NG15 extract, respectively, while ascorbic acid (5 μg/mL) showed 54.86 ± 0.31% of DPPH radical scavenging activity. The DPPH radical scavenging activity at 1000 μg/mL of NG15 extract was slightly higher than that of ascorbic acid (5 μg/mL) (Figure 3a). To investigate the anti-inflammatory activity of the NG15 extract, we analyzed the effect of the extract on TNF-α-induced NF-κB activation by NF-κB-dependent luciferase reporter assay. When the NF-κB luciferase reporter NIH3T3 stable cells were treated with various concentrations of the NG15 extract, TNF-α-induced luciferase activity was decreased in a dose-dependent manner up to 15.40 ± 0.38% at 1000 μg/mL compared to cells treated with only TNF-α (Figure 3b). To determine the protective effect of the NG15 extract on the skin cells after exposure to UVB, the viability of CCD-986sk cells was measured by MTT assay. The viability of CCD-986sk cells after exposure to 30 mJ/cm^2^ of UVB was decreased to 79.01 ± 2.57% of control cells. However, treatment with the NG15 extract significantly reduced the UVB-induced cell death in a dose-dependent manner. The cell viability of CCD-986sk treated with NG15 extract was 87.49 ± 7.49, 89.00 ± 2.89, 97.17 ± 7.19, 100.45 ± 5.62, and 109.48 ± 8.25% after treatment with 100, 250, 500, 750, and 1000 μg/mL of NG15 extract, respectively, compared to control cells (Figure 3c).

### 2.5. Skin Moisturizing and Anti-Wrinkle Activities of NG15 Extract

**Figure 4:**
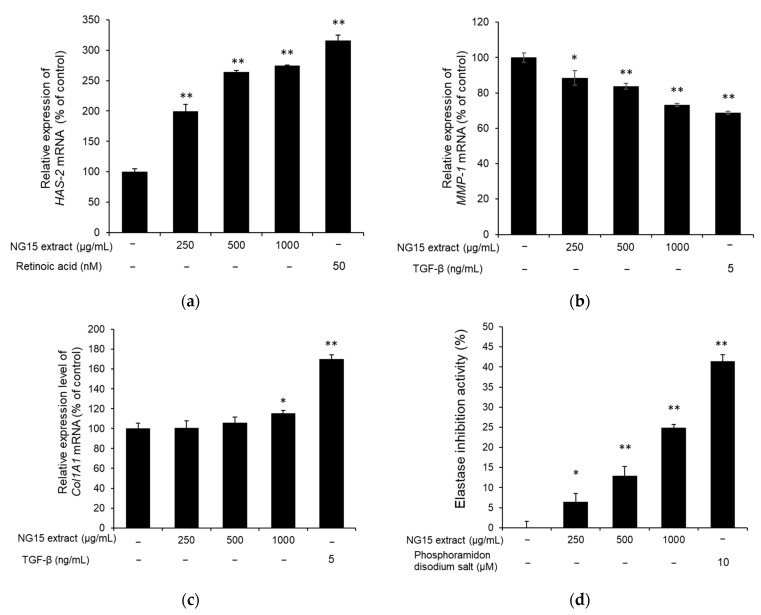
Skin moisturizing and anti-wrinkle activities of NG15 extract. The mRNA expression level of (**a**) *HAS-2*, (**b**) *MMP-1*, and (**c**) *Col1A1*. (**d**) In vitro elastase inhibition activity of NG15 extract against elastase from NHDF cells. * denotes a *p* value < 0.05 versus a normal (untreated) group. ** denotes a *p* value < 0.01 versus a normal (untreated) group. *n* = 3; Student’s *t* test. Retinoic acid (50 nM), TGF-β (5 ng/mL), and phosphoramidon disodium salt (10 μM) were used as a positive control, respectively. The mRNA expression level of each gene was normalized to that of the *β-actin* gene.

To assess the skin hydration activity of the NG15 extract, the expression of *HAS-2* (moisturizing-related gene) in NHDF cells was investigated using qPCR. The *HAS-2* mRNA expression was significantly enhanced by treatment with the NG15 extract in a dose-dependent manner. The relative mRNA expression level of *HAS-2* compared to non-treated cells was 199.88 ± 10.63, 263.91 ± 2.47, and 274.58 ± 1.37%, after treatment with 250, 500, and 1000 μg/mL of the NG15 extract for 24 h, respectively, whereas *HAS-2* mRNA expression level of NHDF cells treated with retinoic acid (50 nM) was 316.31 ± 8.89% (Figure 4a). To determine the effect of the NG15 extract on the collagen degradation and synthesis, the expression of *MMP-1* and *Col1A1* were analyzed using qPCR. The *MMP-1* mRNA expression of the NHDF cells was decreased by treatment with the NG15 extract in a dose-dependent manner, up to 26.71% at 1000 μg/mL, compared to the non-treated control (Figure 4b). The *Col1A1* mRNA expression of the NHDF cells treated with the NG15 extract did not show a statistically significant difference at doses below 500 μg/mL, but it increased by 15.53 ± 2.75% at 1000 μg/mL, compared to the non-treated control (Figure 4c). We also investigated the effect of NG15 extract on the inhibition of elastase activity. The NG15 extract showed elastase inhibitory activity in a dose-dependent manner, up to 24.88 ± 0.80% at 1000 μg/mL, compared to the non-treated control (Figure 4d).

## 3. Discussion

Microalgae are a diverse group of photosynthetic microorganisms. They are considered to be one of the oldest organisms and the original producers of the oxygenic atmosphere on Earth. Since their first appearance over 3 billion years ago, they evolved and diversified by adaptation to survive in a wide range of harsh and extreme environments [46]. They can survive in deserts, volcanic water, acid mine drainage, and in subzero temperatures [47,48]. For example, microalgae can survive desiccation by generating spores to remain dormant under more extreme conditions and then later revive themselves when conditions become normal. The presence of a thick cell wall also helps them to survive by protecting against evaporation [47]. Microalgae have also developed chemical defense systems against reactive oxygen species and free radicals by producing various bioactive compounds including a number of secondary metabolites to protect themselves when they are naturally exposed to oxidative stress, which is mainly caused by UV radiation [28,49]. 

The high-value compounds from microalgae include PUFAs, pigments (chlorophylls, carotenoids, and phycobiliproteins), carbohydrates, peptides, vitamins, polyphenols, and phytosterols [50]. These have diverse biological benefits, including antioxidant, antimicrobial, anti-inflammatory, immunostimulatory, anti-aging, photoprotective, anti-melanogenic, and skin-moisturizing properties [28,50]. Despite the great potential of microalgae for the production of diverse bioactive compounds, only a few species among more than 30,000 microalgal species have been used in some commercial applications, including *Spirulina*, *Chlorella*, *Haematococcus,* and *Dunaliella* [32]. Therefore, it is necessary to explore more microalgal species as candidates for their biotechnological and industrial applications. *Nannochloropsis*, which belongs to eustigmatophycean algae, has desirable properties for cosmeceutical applications due to its suitability for intensive culture and high content of PUFAs (in particular, EPA), carotenoids, polyphenols, and some vitamins [40].

In the present study, we evaluated the potential of the *Nannochloropsis* sp. G1-5 (NG15) strain for cosmeceutical applications, which was isolated from the southern West Sea of the Republic of Korea. We found that the NG15 extract, obtained by ethanol extraction, contained fatty acids, carotenoids, phenolics, and flavonoids. In particular, it has various PUFAs like γ-linolenic, linoleic acid, and EPA, which are known to have anti-aging, photoprotection, and anti-inflammatory activities. It also contained strong antioxidant compounds like astaxanthin, zeaxanthin, canthaxanthin, β-carotene, phenolics, and flavonoids, which can contribute to photoprotection, anti-aging, and anti-melanogenesis. As the NG15 extract contained valuable bioactive compounds with potential for cosmeceutical applications, we further investigated its various functions related to skin protection. Although microalgae have been recognized and exploited as alternative food supplements for animal and human nutrition, they are also potential sources of dangerous toxins because it has been reported that about 2% of 4000 microalgal species can produce neuro- or hepato-toxins with severe cytotoxic effects to human cell lines [40]. Therefore, toxicological bioassay should be considered for the application of microalgal extract for cosmeceutical applications. In this context, we confirmed that the NG15 extract has no significant cytotoxicity up to the concentration of 1000 μg/mL to all tested cell lines including B16F10, CCD-986sk, NHDF, and NF-κB luciferase reporter NIH3T3 stable cells. 

The NG15 extract exhibited tyrosinase inhibition activity and decreased melanin content. Melanin synthesis can be stimulated by α-MSH. The binding of α-MSH to melanocortin 1 receptor (MC1R) leads to the activation of adenylyl cyclase and promotes the generation of cyclic AMP (cAMP). The cAMP accumulation promotes the activation of protein kinase A (PKA), leading to the phosphorylation of the cAMP-responsive binding element (CREB). The activation by CREB increases the expression of microphthalmia transcription factor (MITF) protein and the expression of tyrosinase [51,52]. In this analysis, after induction of melanogenesis with α-MSH in B16F10 cells, we measured the anti-melanogenic effect of the NG15 extract. Melanin content was suppressed in a dose-dependent manner up to 48% in comparison with the α-MSH-only-treated cells. Tyrosinase catalyzes the rate-limiting step of melanogenesis and is widely used as a target to find novel inhibitors of melanin biosynthesis. Therefore, to investigate the mechanism by which the NG15 extract suppress melanogenesis, we analyzed whether the NG15 extract could directly inhibit tyrosinase activity. The NG15 extract significantly decreased the mushroom tyrosinase up to 59.2%, which was higher than arbutin (300 μM) used as a positive control. Our results indicated that the NG15 extract can be applied as an anti-melanogenic agent.

UV radiation is one of the main reasons for free radical (e.g., ROS) production in the skin. The resulting oxidative stress can lead to cell damage and cell death via apoptotic or necrotic processes, which is noticeable with the presence of wrinkles and skin dryness [5,53]. Our results showed that the NG15 extract has 57.36% of DPPH radical scavenging activity, which was higher than that of ascorbic acid (5 μg/mL), indicating that NG15 extract can be a candidate for natural antioxidant ingredients. Furthermore, the treatment with NG15 extract markedly reduced the UVB-induced cell death of CCD-986sk cells. After exposure to UVB radiation, the viability level of cells treated with the NG15 extract at a concentration of 500 and 1000 μg/mL was similar or higher than that of the control (without UV exposure), implying that the NG15 extract can be utilized as a UV protection agent. 

Maintenance of proper skin hydration and collagen content is important for skin health [4]. HA is a glycosaminoglycan with hydrophilic properties and contributes to the hydration and plasticity of the skin. It is biologically synthesized by regulating the expression of hyaluronan synthetases (HASs) [54]. In particular, HAS-2 is highly expressed in vertebrates and plays a crucial role in HA synthesis in comparison with other HASs such as HAS-1 and HAS-3 [55]. Our results showed that treatment with the NG15 extract significantly enhanced the expression of *HAS-2* in a dose-dependent manner up to 274.58% at a concentration of 1000 μg/mL compared to the non-treated cells, indicating the potential of NG15 extract as a skin moisturizing agent. ROS inhibits collagen synthesis and promotes MMP expression, which is a key enzyme in the breakdown of the collagenous extracellular matrix in dermal connective tissues to accelerate the formation of wrinkles and skin aging [56]. MMP-1, interstitial collagenase, initiates the degradation of collagen fibers by cleaving collagen into 3/4 and 1/4 fragments. These fragments then become unfolded and degraded by MMP-2, -9, and -3 [57]. Our results showed that the NG15 extract suppressed the *MMP-1* mRNA expression up to 26.71% at 1000 μg/mL as well as increased *Col1A1* mRNA expression up to 15.53%. In addition, it inhibited elastase activity up to 24.88%. Therefore, our results suggest that the NG15 extract could be a candidate for an anti-winkling agent, suppressing degradation of collagen and elastin and promoting procollagen synthesis. Although a previous study [41] presented the skin protective effect of the extract from *Nannochloropsis gaditana*, there was no investigation on the UV protective effect and expression of hyaluronic acid synthetase, which are essential for skin protection. In comparison to the previous study, we confirmed that the NG15 extract decreased the cell death induced by exposure to UVB and improved the expression of *HAS-2* gene. In addition, the NG15 extract exhibited higher tyrosinase inhibition activity (59.2%) than that (~10%) of the previous study at a concentration of 1000 μg/mL [41]. Furthermore, we found that total phenolics’ and flavonoids’ contents in the crude extract of NG15 were higher than those of other microalgae previously reported [43,44,45].

Recently, there has been emerging interest in various natural products with skin protective effects for cosmeceutical applications. However, some of these natural products are obtained from fish, other aquatic organisms, or higher plants, which are connected with important environmental issues including overfishing and overexploiting of wild medicinal plants and other natural resources. Microalgae have various advantages as a sustainable source for natural bioactive compounds. Microalgae have higher photosynthetic efficiency compared to terrestrial plants (about 3% vs. <1% for higher plants). In addition, they do not require arable land or a supply of fresh water and they can be harvested nearly all year round, thus not compromising the production of food or other products from crops. Compared to terrestrial plants, their high productivity and the ease of cultivation, harvesting, and extraction at a large scale make them attractive for commercial production of bioactive compounds [25,50]. In this study, we found that the extract from *Nannochloropsis* sp. G1-5 had a wide range of skin-protective effects including antioxidant, anti-melanogenic, anti-inflammatory, UV protective, anti-wrinkling, and skin moisturizing activities. These activities might be attributed to PUFAs, carotenoids, and phenolics. Therefore, it is believed that the NG15 extract has potential for the development of natural cosmetics with various skin protective functions. The mechanisms underlying various skin protective effects of the NG15 extract will be fully elucidated in our further studies. Given that the topical application of natural bioactive compounds is limited by low bioavailability associated with their inherent chemical instability and poor solubility, encapsulation of the microalgal extract can be considered as a promising solution for the development of cosmetic products with stable and enhanced bioactivities [58].

## 4. Materials and Methods

### 4.1. Isolation, Identification, and Cultivation of Microalgae

Seawater samples were collected from coastal points of the southern West Sea of Korea. After spreading the seawater sample onto an F/2 agar plate, a colony with brownish color was isolated and grown in the liquid F/2 medium [59] at 23 °C, 50 μmol photons/m^2^/s. Genomic DNA was extracted using a method described previously [60]. Partial 18S ribosomal DNA was PCR amplified using primers (Table 4), and the sequence was analyzed using BLAST. The cultivation of *Nannochlroropsis* sp. G1-5 was performed using a modified F/2 medium with a double concentration of NaNO_3_ and NaH_2_PO_4_·2H_2_O. The composition of the modified F/2 medium was as follows: 150 mg NaNO_3_, 11.3 mg NaH_2_PO_4_·2H_2_O, 4.16 mg Na_2_EDTA, 3.15 mg FeCl_3_·6H_2_O, 0.01 mg CuSO_4_·5H_2_O, 0.022 mg ZnSO_4_·7H_2_O, 0.01 mg CoCl_2_·6H_2_O, 0.18 mg MnCl_2_·4H_2_O, 0.006 mg Na_2_MoO_4_·2H_2_O, 0.1 mg Thiamine HCl, 0.5 μg Cyanocobalamin, and 0.5 μg Biotin in 1 L artificial sea water. The cells were grown in a 1-L Erlenmeyer flask with a working volume of 300 mL at 23 °C, 80 μmol photons/m^2^/s, agitation rate of 115 rpm, and 5% CO_2_ in a CO_2_ incubator for 10 days. Cell growth was monitored by measuring the optical density at 800 nm (OD_800_) with a spectrophotometer (Eppendorf), setting the initial OD_800_ to 0.2. The cells were then transferred to the modified F/2 medium supplemented with an additional 27 g/L of NaCl and cultured for 20 days under the same conditions. Then, 30 mL of cell culture was harvested by centrifugation at 5000× *g* for 15 min and the cell pellet was stored at −80 °C.

### 4.2. Preparation of the NG15 Extract

The frozen cell pellet obtained from 30 mL of culture was extracted with 15 mL of absolute ethanol by vortexing at a maximum speed for 15 min two times at room temperature. The supernatant was filtered using a PTFE syringe filter (pore size of 0.2 μm) to remove cell debris. The extraction solvent was evaporated using a rotary vacuum evaporator and centrifugal evaporator to yield dried extract.

### 4.3. Analysis of Carotenoids and Fatty Acid Methyl Esters

The carotenoids in the crude extract of NG15 were analyzed using a HPLC system (1260 infinity, Agilent, Santa Clara, CA, USA). Analytical separations were performed using a Horizon C18/PFP column, 150 mm × 4.6 mm, and 3-μm particle size (Horizon Chromatography, Halifax, UK) by a previously described method [61]. Chlorophyll a and carotenoids were detected by diode-array spectroscopy (300–720 nm). LC-MS analysis was carried out using an Agilent 1290 infinity II HPLC system with a diode array detector and ISQ EC mass spectrometer (Thermo Fisher Scientific, Waltham, MA, USA) with a HESI-II electrospray ionization source. The separation of carotenoids and chlorophyll a was performed using a Horizon C18/PFP column, 150 mm × 4.6 mm, and 3-μm particle size as described above except that 0.1% (*v*/*v*) formic acid was added to mobile phase solvents. MS analysis was performed using parameters as follows: vaporizer temperature = 317 °C; ion transfer tube temperature = 350 °C; optimum sheath gas pressure = 58.8 psig; auxiliary gas pressure = 5.2 psig; sweep gas pressure = 2 psig; and positive mode with source voltage of 4 kV and variable collision-induced dissociation (CID) voltage. Identification was performed based on the comparison of the retention times and UV-visible and mass spectral characteristics of peaks with those of standards and data available in the literature [61,62,63,64]. The pigments were quantified using standard curves of standard pigments (Sigma-Aldrich, St. Louis, MO, USA) or specific absorption coefficient for vaucheriaxanthin (232 L/g/cm) and its ester (182 L/g/cm) [65].

FAME (fatty acid methyl ester) was prepared by acid-catalyzed transesterification of the crude extract of NG15. Two milliliters of methanolic sulfuric acid (3%, *v*/*v*) were added to a 15-mL, screw-capped, glass tube containing the total lipid extract in 1 mL of hexane. The mixture was vortexed and heated at 95 °C for 1.5 h. After cooling, 2 mL of water and hexane were added, and FAMEs were separated by collecting the organic phase. The extracted FAMEs were analyzed using a gas chromatograph (7890A GC, Agilent Santa Clara, CA, USA) equipped with a flame ionization detector and a DB-FastFAME column, 30 m × 0.25 mm, and 0.25 µm (Agilent, Santa Clara, CA, USA) with the following conditions: injection volume 1 μL; split ratio 1:50; injector temp 250 °C; detector temp. 280 °C; and oven temperature, held at 50 °C for 0.5 min, increased to 194 °C at 30 °C/min, and increased to 240 °C at 5 °C/min. FAMEs were identified and quantified by retention time and comparison with FAME standards (Sigma-Aldrich, St. Louis, MO, USA).

### 4.4. Determination of Total Phenolic and Total Flavonoid Content

The total phenolic content of the NG15 extract was analyzed by colorimetric Folin–Ciocalteu method as described previously [66]. Briefly, a 100-μL sample or gallic acid solution, which dissolves in methanol at different concentrations, was mixed with 200 μL of 10% Folin–Ciocalteu reagent. Then, 800 μL of Na_2_CO_3_ (700 mM) was added, and the mixture in assay tubes was incubated at 25 °C for 2 h. Finally, 200 μL of the mixture was transferred to a 96-well microplate, and the absorbance was quantified by a microplate reader (Spectramax i3x, Molecular Devices, San Jose, CA, USA) at 765 nm. Gallic acid was used as the reference standard. The results were expressed as mg gallic acid equivalents (GAE)/g dry weight (DW). Total flavonoid content was determined using the classical colorimetric method as described previously [66]. Briefly, 100 μL of 2% aluminum trichloride (AlCl_3_) in methanol was mixed with the same volume of the sample extracts. Absorption readings at 415 nm were taken after 10 min against a blank sample consisting of a 100 μL of extract solution with 100 μL of methanol without AlCl_3_. The calibration curve was prepared using various concentrations of quercetin (0–250 μg/mL) dissolved in methanol solution. The results were shown as mg quercetin equivalent (QE)/g DW.

### 4.5. Cell Culture

The B16F10 murine melanoma cells (ATCC, Manassas, VA, USA), CCD-986sk cells (KCLB, Seoul, Korea), and NF-κB luciferase reporter NIH-3T3 stable cells (Panomics, Fremont, CA, USA) were cultured in Dulbecco’s Modified Eagle’s Medium (DMEM), supplemented with 10% fetal bovine serum (FBS), 100 units/mL of penicillin, and 100 μg/mL of streptomycin in culture flasks in a CO_2_ incubator with a humidified atmosphere of 5% CO_2_ in air at 37 °C. The Normal Human Dermal Fibroblast cells (NHDF, CELLnTEC, Bern, Switzer-land) were cultured in Fibroblast Basal Medium (Lonza, Hayward, CA, USA), supplemented with FGM-2 singleQuots (hGFG, insulin, FBS and gentamicin/amphotericin-B; Lonza, Hayward, CA, USA) in culture flasks in a CO_2_ incubator with a humidified atmosphere of 5% CO_2_ in air at 37 °C.

### 4.6. Cell Viability Assay

The B16F10 cells were seeded at a density of 1.5 × 10^4^ cells per well in a 96-well plate and incubated for 24 h, then treated with the NG15 extract for 72 h in the medium without FBS. The NHDF cells were seeded at a density of 4 × 10^3^ cells per well in a 96-well plate and incubated for 24 h, then treated with the NG15 extract for 24 h in the medium without FBS. NF-κB luciferase reporter NIH3T3 stable cells were seeded at a density of 3 × 10^3^ cells per well in a 96-well plate and incubated for 24 h, then treated with the NG15 extract for 24 h in the medium without FBS. CCD-986sk cells were seeded at a density of 1 × 10^4^ cells per well in a 96-well plate and incubated for 24 h, then treated with the NG15 extract for 24 h in the medium without FBS. Cell viability for the previously mentioned cell lines was measured using the WST-1 assay (B16F10, NHDF, and NF-κB luciferase reporter NIH3T3 stable cells) or MTT assay (CCD-986sk). For WST-1 assay, each well with cultured cells was supplemented with 10% (*v*/*v*) of WST-1 assay solution (EZ-CYTOX; DOGEN, Seoul, Korea) and incubated for 1 h. The absorbance was measured at 450 nm by a microplate reader (Spectramax i3x, Molecular Devices, San Jose, CA, USA). For MTT assay, MTT solution (5 mg/mL) was added and incubated for an additional 4 h. The medium was removed, and 100 μL of dimethyl sulfoxide were supplied to dissolve formazan. The absorbance was measured at 570 nm using a microplate reader (Spectramax i3x, Molecular Devices, San Jose, CA, USA).

### 4.7. Determination of Antioxidant Activity

The DPPH radical scavenging activity was evaluated according to the method described previously with modifications [67]. Twenty microliters of the NG15 extract (0–1000 μg/mL) were mixed with 120 μL of 1 mM DPPH solution. The reaction was then incubated for 30 min at room temperature under dark condition. The absorbance was measured at 517 nm by a microplate reader (Spectramax i3x, Molecular Devices, San Jose, CA, USA). L-Ascorbic acid (BioXtra, ≥99.0%, Sigma-Aldrich, St. Louis, MO, USA) was used as a positive control. DPPH radical scavenging activity was calculated by the following equation:

DPPH radical scavenging activity (%) = [1 − ((A1 − A2))/A0] × 100, (1)

where A0 is the absorbance of the DPPH solution without the sample, A1 is the Absorbance of the DPPH solution with the sample, and A2 is the absorbance of the sample without the DPPH solution.

### 4.8. Determination of Tyrosinase and Elastase Inhibition Activity

For determining the tyrosinase inhibition activity, 2 mM L-tyrosine (50 mM Potassium phosphate buffer, pH 6.8) and the NG15 extract (0–1000 μg/mL) were incubated for 30 min with mushroom tyrosinase (1000 U/mL) at 37 °C. The absorbance of each sample was measured at 490 nm by a microplate reader (Spectramax i3x, Molecular Devices, San Jose, CA, USA). Arbutin (300 μM) was used as a positive control. The tyrosinase inhibition activity was calculated by the following equation:

Tyrosinase inhibition activity (%) = [1 − ((A1 − A2))/A0] × 100, (2)

where A0 is the absorbance of the tyrosine solution without the sample, A1 is the Absorbance of the tyrosine solution with the sample, and A2 is the absorbance of the sample without the tyrosine solution.

For determination of elastase inhibition activity, NHDF cells washed with PBS were lysed with 0.1% triton X-100 (0.2M Tris-HCl, pH 8.0) and homogenized using a sonicator. After centrifugation, the supernatant was used as crude elastase. The NG15 extract and crude elastase were mixed with 50 mM N-succinyl-tri-alanyl-p-nitroanilide (STANA), and the reaction was incubated for 90 min at 37 °C. The absorbance was measured at 410 nm using a microplate reader (Spectramax i3x, Molecular Devices, San Jose, CA, USA). Phosphoramidon disodium salt (10 μM) was used as a positive control. The elastase inhibition activity was calculated by the following equation:

Elastase inhibition activity (%) = [1 − A1/A0] × 100, (3)

where A0 is the absorbance of the elastase solution without the sample and A1 is the absorbance of the elastase solution with the sample.

### 4.9. Determination of Melanin Content

The B16F10 cells were seeded at a density of 1.5 × 10^5^ cells in a 60-mm culture dish and incubated for 24 h. The cells were treated with the NG15 extract (0–1000 μg/mL) or arbutin (300 μM, positive control) in the presence or absence of α-MSH (100 nM) for 72 h. At the end of the treatment, the cells were lysed with 1N NaOH and centrifuged at 12,000 rpm for 10 min. The absorbance of the supernatant was measured at 490 nm using a microplate reader (Spectramax i3x, Molecular Devices, San Jose, CA, USA).

### 4.10. Determination of Anti-Inflammatory Activity

NF-κB reporter NIH-3T3 stable cells were seeded at a density of 2 × 10^5^ cells per well in a six-well plate and incubated for 24 h. The cells were treated with the NG15 extract (0–1000 μg/mL) for 24 h and then treated with TNF-α (50 ng/mL) for an additional 8 h. After treatment, the cells were harvested and lysed with passive lysis buffer (Promega, Madison, WI, USA). After aliquoting 80 μL of the supernatant containing the same amount of protein in each black 96-well plate, luciferin (Promega, Madison, WI, USA) was added to each well, and luminescence was measured using a microplate reader (Spectramax i3x, Molecular Devices, San Jose, CA, USA).

### 4.11. Determination of Cell Viability after UV Radiation

CCD-986sk cells were seeded at a density of 1 × 10^4^ cells per well in a 96-well plate and cultured for 48 h. After the removal of the medium, the cells were washed twice with PBS. Next, the cells were resuspended in PBS containing the NG15 extract (0–1000 μg/mL) and exposed to UVB (312 nm, 30 mJ/cm^2^) using a UV lamp (Vilber Lourmat, Collégien, France). After the removal of PBS and the NG15 extract, the cells were cultured in the DMEM medium for 24 h. Then, the MTT solution (5 mg/mL) was added and incubated for an additional 4 h. The medium was removed, and 100 μL of dimethyl sulfoxide was supplied to dissolve formazan. The absorbance was measured at 570 nm using a microplate reader (Spectramax i3x, Molecular Devices, San Jose, CA, USA).

### 4.12. Quantitative Real-Time PCR

To analyze the gene expression levels of *HAS-2*, *Col1A1*, and *MMP-1* quantitatively within NHDF cells, we applied the quantitative real-time PCR (qRT-PCR) method. Total RNA was extracted from the cells using a TRIzol reagent (Invitrogen, Waltham, MA, USA) and cDNA was synthesized using M-MLV reverse transcriptase (Invitrogen). The qRT-PCR was performed using SYBR Green Master Mix (Applied Biosystems, Waltham, MA, USA) on a StepOnePlus real-time PCR system (Applied Biosystems, Waltham, MA, USA). The mRNA expression levels of *HAS-2*, *Col1A1*, and *MMP-1* were calculated using the StepOnePlus systems software (Applied Biosystems, Waltham, MA, USA) and normalized to that of the *β-actin* gene. The primers used for qRT-PCR are listed in Table 4.

### 4.13. Statistical Analysis

All data are presented as the mean and standard deviation of at least three independent experiments. All statistical analyses were based on paired Student’s *t* test using Excel. A *p*-value < 0.05 was considered statistically significant.

## 5. Conclusions

In the present study, we explored the cosmeceutical potential of the extract from *Nannochloropsis* sp. G1-5 strain, which was isolated from the southern West Sea of Korea. We found that the ethanol extract from *Nannochloropsis* sp. G1-5 contained PUFAs (including EPA), carotenoids (astaxanthin, canthaxanthin, β-carotene, zeaxanthin, violaxanthin), and phenolic compounds, which are known to have various bioactivities. Based on its content of bioactive compounds, we further analyzed and confirmed that the NG15 extract showed a wide range of skin protective functions with low cytotoxicity, including antioxidant, anti-melanogenic, anti-inflammatory, UV protective, anti-wrinkling, and skin moisturizing activities. Our results indicate that the extract of *Nannochloropsis* sp. G1-5 has the potential to be used for the development of natural cosmetics with a broad range of skin protective functions as an economically viable and sustainable production platform.

## Figures and Tables

**Table 1:** Fatty acid composition of the NG15 extract.

FAME Component	Content (mg/g Extract)
Myristic acid (C14:0)	22.93 ± 0.21
Palmitic acid (C16:0)	215.85 ± 2.80
Palmitoleic acid (C16:1 ω7)	188.95 ± 2.56
Stearic acid (C18:0)	7.56 ± 0.02
Oleic acid (C18:1 ω9)	91.40 ± 1.22
Linoleic acid (C18:2 ω6)	4.63 ± 0.06
γ-Linolenic acid (C18:3 ω6)	2.21 ± 0.03
Eicosatrienoic acid (C20:3 ω6)	1.10 ± 0.02
Arachidonic acid (C20:4 ω6)	16.02 ± 0.25
Eicosapentaenoic acid (C20:5 ω3)	31.53 ± 0.58
Sum	582.19 ± 7.70

**Table 2:** Carotenoid composition of the NG15 extract.

Carotenoid Component	Content (mg/g Extract)
Vaucheriaxanthin	0.82 ± 0.02
Violaxanthin	1.81 ± 0.04
Astaxanthin	0.78 ± 0.02
Zeaxanthin	0.13 ± 0.00
Canthaxanthin	1.93 ± 0.04
Chlorophyll a	5.39 ± 0.11
β-Carotene	5.28 ± 0.21
Sum	16.13 ± 0.44

**Table 3:** Total phenolic and flavonoid content of the NG15 extract.

Total Phenolic Content (mg GAE/g Extract)	Total Flavonoids Content (mg QE/g Extract)
77.29 ± 1.25	20.15 ± 0.28

**Table 4:** Sequences of primers used in this study.

Gene	Forward Primers (5′-3′)	Reverse Primers (5′-3′)
18S rDNA	CCTGGTTGATCCTGCCAGTA	ACCTTGTTACGACTTCTCCTTC
*COL1A1*	AGGGCCAAGACGAAGACATC	AGATCACGTCATCGCACAACA
*HAS-2*	GAAAGGGCCTGTCAGTCTTATTT	TTCGTGAGATGCCTGTCATCACC
*MMP-1*	TCTGACGTTGATCCCAGAGAGCAG	CAGGGTGACACCAGTGACTGCAC
*β-actin*	GGATTCCTATGTGGGCGACGA	CGCTCGGTGAGGATCTTCATG

## Data Availability

The data used to support the findings of this study are available from the corresponding author upon request.

## Supplementary Materials

The following are available online at https://www.mdpi.com/article/10.3390/md19120690/s1. Figure S1: HPLC chromatogram of ethanol extract from *Nannochloropsis* sp. G1-5 (NG15). Table S1: LC-PDA-ESI+-MS analysis of pigments of NG15 extract.
