# Supplementary material for: Exploring the Potential of Nannochloropsis sp. Extract for Cosmeceutical Applications
Source: Mar Drugs. 2021 Dec 2;19(12):690. doi: 10.3390/md19120690 (PMC8704537; doi:10.3390/md19120690)
Supplement: Supplementary file 1 [file marinedrugs-19-00690-s001.zip › marinedrugs-1486808-supplementary.pdf]

Supplementary Materials

Exploring the potential of *Nannochloropsis* sp. extract for cosmeceutical applications

Sun Young Kim, Yong Min Kwon, Kyung Woo Kim and Jaoon Young Hwan Kim

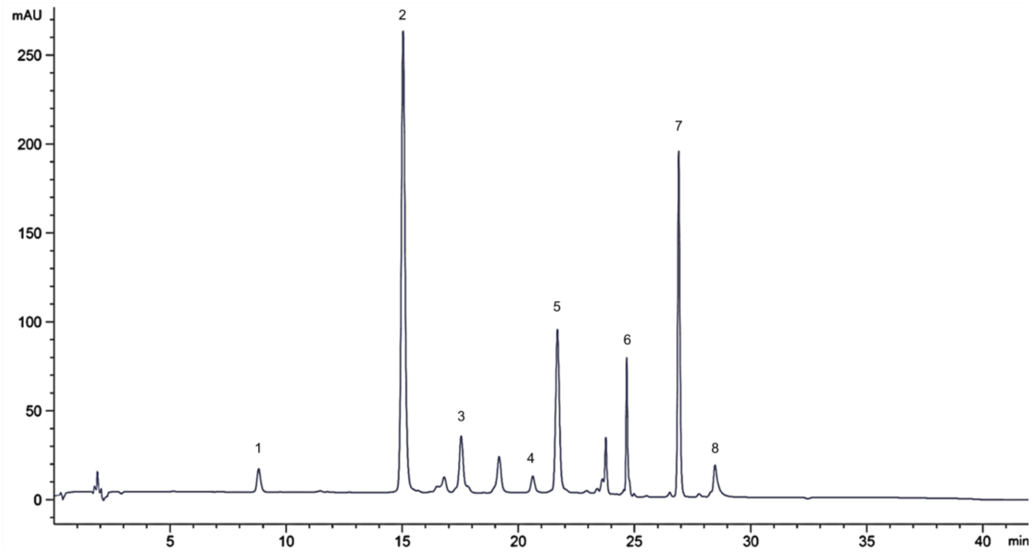

Figure S1. HPLC chromatogram of ethanol extract from *Nannochloropsis* sp. G1-5 (NG15).

Table S1. LC-PDA-ESI$^+$-MS analysis of pigments of NG15 extract.

Peak	t_R (min)	Name	λ_{max} (nm)	ESI$^+$-MS (*m*/*z*)	References
1	8.82	Vaucheriaxanthin	417, 441, 470	618.32 ($[M+H]^+$)	61,62
2	15.04	Violaxanthin	417, 441, 470	601.39 ($[M+H]^+$)	61–63
3	17.54	Astaxanthin	482	597.38 ($[M+H]^+$)	61–63
4	20.62	Zeaxanthin	453, 478	568.68 ($[M]^+$)	61–63
5	21.68	Vaucheriaxanthin ester	422, 445, 473	785.71 ($[M+H]^+$)	61,62
6	24.67	Canthaxanthin	478	565.48 ($[M+H]^+$)	61–63
7	26.90	Chlorophyll a	431, 664	893.4 ($[M+H]^+$)	64
8	28.47	β-carotene	452, 479	536.17 ($[M]^+$)	61–63
